# Natural Alpha-Glucosidase Inhibitors Rapid Fishing from Cyperus Rotundus Using Immobilized Enzyme Affinity Screening Combined with UHPLC-QTOF MS

**DOI:** 10.22037/ijpr.2019.1100753

**Published:** 2019

**Authors:** Shiren Deng, Linbo Xia, Xiamin Zhu, Jiang Zhu, Mingchen Cai, Xin Wang

**Affiliations:** *College of Pharmacy, Liaoning University of Traditional Chinese Medicine, Dalian 116600, China.*

**Keywords:** Affinity fishing, Alpha-glucosidase inhibitors, Immobilized enzyme, Cyperus rotundus, Stilbenoid timers, UHPLC-QTOF MS

## Abstract

An efficient and rapid affinity-based screening method for directly fishing out natural alpha-glucosidase inhibitors from *Cyperus rotundus* extract by using immobilized enzyme technology combined with UHPLC-QTOF MS analysis was established. As a result without time-consuming and laborious isolation workload and false positive interference, five natural alpha-glucosidase inhibitors were successfully recognized and identified from only 400 uL of C. rotundus extracts within only a couple of hours, which suggested that the screening method was rapid, economical, sensitive and feasible. In addition, the captured compounds were isolated and characterized as stilbenoids oligomers, and were proved to be strong alpha-glucosidase inhibitors by inhibitory assay *in-vitro*. Among them, 3 stilbenoids trimers were reported to be potent α-glucosidase inhibitors for the first time. This method could be modified and have the potential for rapidly screening of active compounds extracts against some new targets by immobilizing some other biomacromolecules.

## Introduction

α-Glucosidase inhibitors (AGHI), which have been proved to inhibit α-glucosidase presented in the brush border of the small intestine and consequently delay the digestion and absorption of carbohydrates, are usually used in the treatment of type 2 diabetes mellitus (1). Although strong synthetic AGHI (i.e., acarbose) are available, they usually cause significant adverse events (2). So, exploring some novel AGHI was always an important and emergency work in diabetes researches (3). Natural products, especially some Chinese herbal medicines (such as *Cyperus rotundus* L., Xiang fu-zi in Chinese), were reported to treat diabetes for a long-time in China and considered to be an effective and available source of novel drug candidates (4, 5). Nowadays, affinity screening technology has been considered to be an efficient and rapid method for directly fishing out highly-affinity ligand from complex mixtures (6-10). Some rapid and viable affinity screening models, which can fish out and characterize synthesized and naturally active components directly from real biology samples by using immobilized biomolecules (such as enzyme, receptor and DNA, etc.) technology combined with UHPLC-MS analysis were established (6-10). In this study, over 20 kinds of Chinese herbs were investigated for their α-glucosidase inhibitory. Among them, roots of *Cyperus rotundus* L. showed the best activity. To rapidly fishing out the natural anti-α-glucosidase ingredients, the extract of *C*.* rotundus* was used as the real biological sample to carry out the affinity screening procedure.

## Experimental

*General Experimental Procedures*

Agilent 1290 Infinity LC-6520 Accurate-Mass QTOF-MS (Agilent Technologies, PaloAlto, CA, USA); Bruker DRX-400 (400MHz for ^1^H and 100MHz for ^13^C) spectrometer (Bruker, Rheinstetten, Germany); *α*-Glucosidase (E.C. 3.2.1.20) from *Saccharomyces cerevisiae*, p-nitrophenyl a-D-glucopyranoside (PNPG) and glutathione (GSH) were purchased from Sigma-Aldrich (St Louis, MO, USA). CNBr-activated Sepharose^TM^ 4B was purchased from Phamacia Biotech AB (Uppsala, Sweden). Distilled water was purified by a Milli-Q water purification apparatus (Millipore, Bedford, MA). All other reagents were of analytical grade.

*Plant material*

20 kinds of traditional Chinese herbs (including *C.* *rotundus*) were collected from Yangguang drugstore, Dalian, China. The specimens were identified by Prof. Zhai Yanjun, Liaoning University of Traditional Chinese Medicine, China. Voucher specimens were deposited at College of Pharmacy, Liaoning University of Traditional Chinese Medicine (Voucher No. from LNU-DSR-2015-1 to LNU-DSR-2015-26).

*Immobilization of a-glucosidase*

*a-*Glucosidase (0.20 mg) was dissolved in coupling buffer (0.1 M NaHCO_3_ and 0.5 M NaCl at pH 8.3) at a concentration of 2.0 mg protein/mL. The protein solution was mixed with CNBr-activated Sepharose^TM^ 4B gel at room temperature in a ratio of 100 L protein solution to 5 mg Sepharose^TM^ powder, which would swell to about 20 mL of wet gel. The gel slurry was rotated at room temperature for 1 h to ensure the enzyme bound to the beads. After protein coupling, the resident reactive sites on the gel were blocked by reacting with 0.01 M Tris-HCl buffer at pH 8.0 for 1 h at room temperature. After alternatively washed with 0.1 M CH_3_COONa buffer (pH 4.0 containing 0.5 M NaCl) and 0.1 M Tris-HCl buffer (pH 8.0 containing 0.5 M NaCl), the gel was kept in affinity buffer for further use. Control supports were prepared in the same manner but with no AGH being added during the immobilization step.

Enzyme immobilization yields were determined by comparison of the amount of free enzyme in the solutions before and after coupling to the gel according to the method described by Bradford using BSA as standard protein, giving 9.5 mg AGH/mL gel.

The specific activity of immobilized AGH was determined as 18.4 U/mg protein (175 U/mL gel).

*Preparation of affinity solution of C. rotundus*

200 mg ground *C. rotundus* powder was extracted in 20 mL of 80% aqueous methanol by ultrasonic extraction for 30 min. The extract was filtered and freeze-dried, then redissolved in 10 mL affinity buffer (67 mM KH_2_PO_4_ (containing 5% methanol) at pH 6.8) and filtered through a 0.45 μm membrane as the tested extract sample.

*AGH inhibitors screening by immobilized AGH affinity fishing*

*C. rotundus* extracts (400 mL) was mixed with 20 mL immobilized AGH gel and gently stirred at 37 °C for 20 min. After incubation with the extracts, the gel were washed with 400 mL affinity buffer (67 mM KH_2_PO_4_ (containing 5% methanol) at pH 6.8) four times to remove any unbound components and then treated with 200 mL desorbed solution (50% methanol aqueous solution at pH 3.3) to release the captured potential inhibitors of AGH. The released components were collected and concentrated to 10 mL under N_2_ atmosphere at 40 ºC, then applied to UPLC-QTOF-MS for peak identification. Elution from the control supports was collected and analyzed in the same manner.

*UHPLC-QTOF-MS analysis*

UHPLC-MS was performed on an Agilent 1290 Infinity LC-6520 Accurate-Mass QTOF-MS (Agilent Corporation, CA, USA). Analytes eluted from the AGH coupling supports and control supports were separated on a poroshell 120 EC-C_18_ column (3.0 × 50 mm, 2.7 µm , Agilent Corporation, MA, USA). The solvent system consisted of solvent A (menthol) and solvent B (0.5% formic acid in water, v/v). The solvent A content of the mobile phase was gradient increased from 5% to 100% linearly in 20 min. 

**Table 1:** Inhibitory of 20 species of traditional Chinese herbal on *α*-glucosidase

**Scientific name**	**Parts**	**Inhibitory (%)**	
		**0.1 mg/mL**	**0.5 mg/mL**
*Astragalus membranaceus *(Fisch.) Bunge var. *mongholicus *(Bunge) P. K. Hsiao	Root	1.2	5.1
*Salvia miltiorrhiza *Bunge.	Root	4.5	8.0
*Rheum palmatum *L.	Root	3.0	12.6
*Taraxacum mongolicum *Hand.-Mazz.	Herb	5.6	19.2
*Schisandra chinensis *(Turcz.) Baill.	Fruit	5.0	25.5
*Trigonella foenum-graecum *L.	Seed	15.0	38.5
*Cinnamomum cassia *Presl	Bark	19.6	40.0
*Cuscuta chinensis *Lam.	Seed	14.5	40.1
*Atractylodes lancea *(Thunb.) DC.	Root	9.4	40.5
*Aucklandia lappa *Decne	Root	25.7	43.4
*Angelica sinensis *(Oliv.) Diels	Root	22.5	47.0
*Pueraria lobata *(Willd.) Ohwi	Root	14.0	48.6
*Anemarrhena asphodeloides *Bunge	Root	20.0	48.8
*Gardenia jasminoides *Ellis	Fruit	40.0	72.8
*Peonia lactiflora *Pall.	Root	43.8	81.0
*Canavium album *Raeusch.	Fruit	74.5	87.9
*Morus alba *L.	Root-bark	86.9	93.3
*Sophora japonica *L.	Flower	92.8	97.9
*Glycyrrhiza uralensis *Fisch. Ex DC.	Root	81.6	98.7
*Cyperus rotundus *L.	Root	95.1	99.5

**Table 2:** Inhibitory effects of stilbenoids and reference on alpha-glucosidase activities

**Compounds**	**alpha-glucosidase inhibitory**	
	**IC** **50 ** **(µM)**	**Kinetic mode (** ***K*** ***i*** **, µM)**
Cyperusphenol A **(1)**	1.43 ± 0.11	Noncompetitive (11.2)
Mesocyperusphenol A **(2)**	1.44 ± 0.17	Noncompetitive (10.7)
Cyperusphenol D **(3)**	1.18 ± 0.11	Noncompetitive (8.63)
Scirpusins B **(4)**	13.3 ± 1.66	Noncompetitive (95.4)
Scirpusins A **(5)**	21.4 ± 1.40	Noncompetitive (136)
Acarbose (reference)	1470 ± 117	competitive (834)

**Scheme 1:**
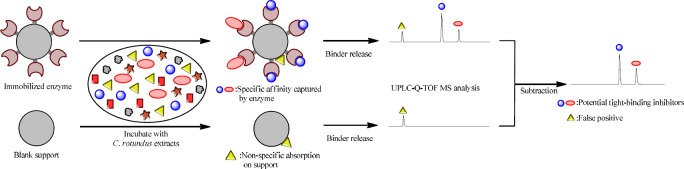
Rapid screening of natural AGHI from *C*. *rotundus *by affinity capture system

**Figure 1:**
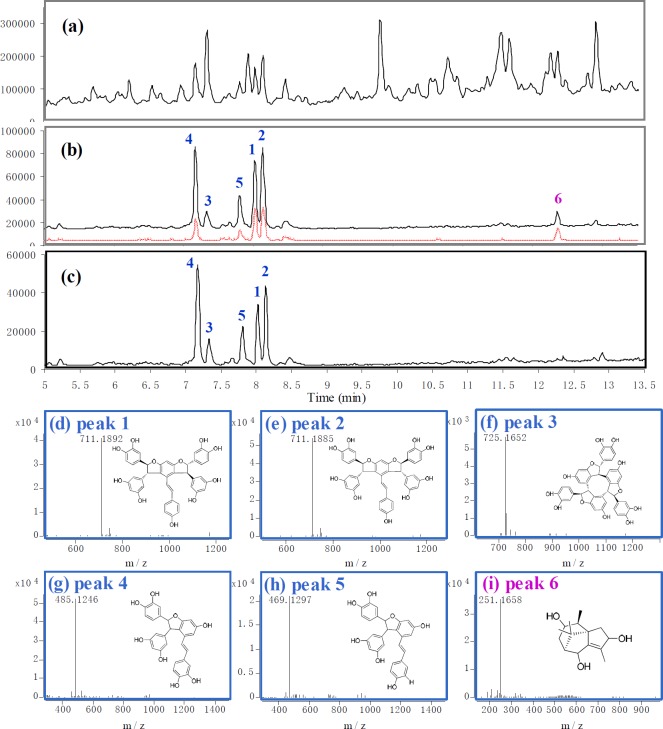
Screening results of *C*. *rotundus *by using affinity capture method. (a) TIC of *C*. *rotundus *extract. (b) TIC of the binders screened out from *C*. *rotundus *extract. The black solid line represents the binders absorbed on immobilized α-glucosidase. The red dotted line represents the binders absorbed on blank supports (false positive). (c) TIC of the highly-affinity ingredients, which was made by subtracting the signal of false positive. (d)-(i) The accurate mass spectrum and structure of peak 1-6 (cyperusphenol A, mesocyperusphenol A, cyperusphenol D, scirpusins B, scirpusins A, sugetriol)

**Figure 2:**
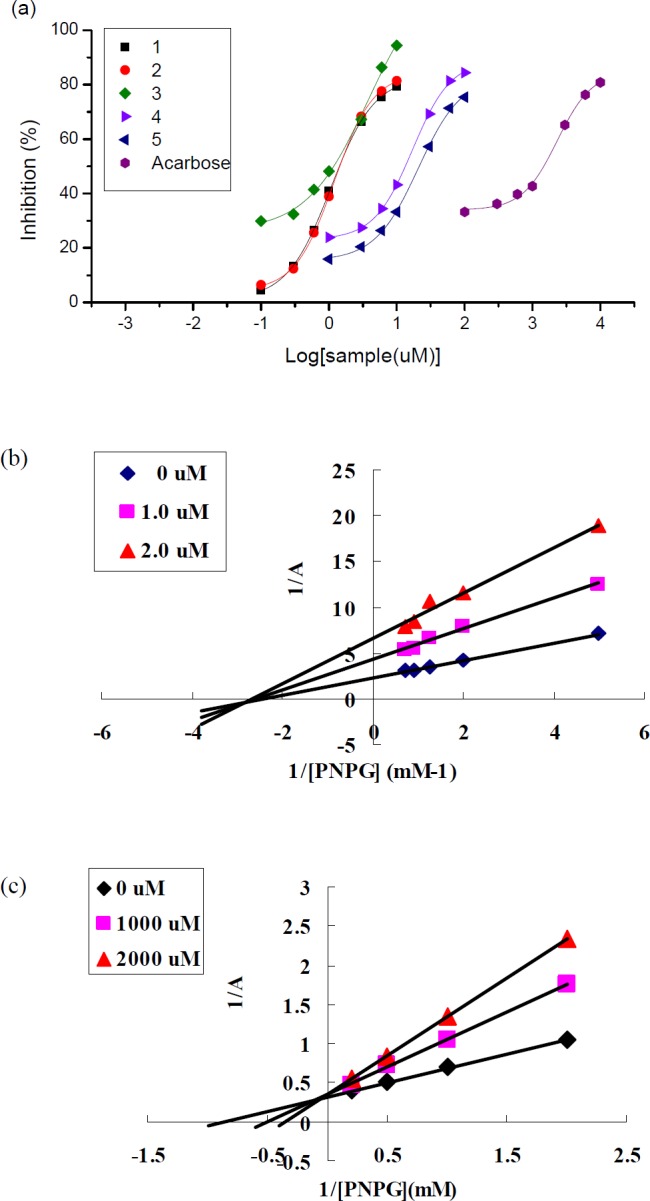
Inhibitory effects of compounds 1-5 on  -glucosidase (a) and Lineweaver-Burk plots of compound 1(b) and acarbose(c)

The flow rate was set at 0.4 mL/min. Mass analysis was performed in negative ESI ion mode. The operating parameters were as follows: drying gas (N_2_) flow rate, 8 L/min; drying gas temperature, 325 ^o^C; nebulizer, 30 psig; capillary, 3500 V; skimmer, 65 V; fragmentor voltage, 100 V. The mass range was set at m/z 100–2000. The system was operated under Agilent MassHunter workstation acquisition software, version B.02.01 (Agilent Corporation, MA, USA).

*Isolated procedure of 5 natrual AGHIs from C. Rotundus*

The shade dried *C. rotundus *(1 kg) was powdered and extracted with 80% aqueous methanol (3 × 2 L) by ultrasonic extraction for 60 min at room temperature. The solution was filtered and concentrated under reduced pressure on a rotatory evaporator, yielding 102 g of crude extract. The entire methanol crude extract (102 g) was suspended in 1 L of water and then partitioned sequentially with equal volumes of *n*-hexane, EtOAc, and *n*-BuOH. The methanol extract of four fractions were detected by UHPLC-MS, respectively. Amnog them, EtOAc fraction (23 g) was found to have most of the stilbenoids oligomers and was separated over silica gel. The EtOAc fractions were eluted with *n*-hexane, CH_2_Cl_2_, CH_2_Cl_2_/MeOH (9:1 ® 7:3 ® 5:5 ® 3:7 ® 1:9 ® only MeOH) to give eight fractions (F1-F8). F4 (70% CH_2_Cl_2_, 2.8 g) was found to be the aboundent part by LC-MS. Then the F4 fraction was subjected on Prep-RP-HPLC with 35% aqueous MeOH as an elunt to give compound **1 **((E)-cyperusphenol A**,** 24 mg), **2 **(mesocyperusphenol A, 11 mg), **3 **(Cyperusphenol D, 6.6 mg), **4** (Scirpusins B, 19 mg) and **5 **(Scirpusins A, 65 mg).

*Preparation of crude extracts of 20 herbs*

Each of the dried ground powder (3 g) was extracted in 30 mL of 80% aqueous methanol by ultrasonic extraction for 60 min at room temperature. After centrifugation at 5000 rpm for 5 min, the combined extract was evaporated under reduced pressure to dryness. To each herb extract, DMSO was added to get 5 mg/mL solution, respectively.

*α-Glucosidase inhibitory activity assay in vitro and kinetics analysis*

The *α*-glucosidase inhibitory activity was determined spectrophotometrically on 96-well microplate reader. In brief, to a total of 310 μL of reaction mixture containing 265 μL of 67 mM phosphate buffer (pH 6.8), 10 μL of 3 mM glutathione, 25 μL of 10 mM *p*-nitrophenyl-*α*-D-glucopyranoside and 10 μL of investigated compounds in the wells was added 10 μL of 0.3 U/mL *α*-glucosidase and mixed. All the reagents used in this assay were dissolved in the same phosphate buffer (67 mM, pH 6.8). After incubation for 10 min at 37 oC, the reaction was stopped by adding 800 μL of 0.1 M Na_2_CO_3 _solution. Then the absorbance of sample (AS) at 405 nm was recorded (SunriseTM microplate absorbance reader, Tecan, Austria). The control was the same mixture except for the investigated sample replaced by the phosphate buffer. The sample blank and control blank were the same mixtures as sample and control, respectively, except *α*-glucosidase was instead with phosphate buffer, respectively.

The *α*-glucosidase inhibition activity (%) of test sample could be calculated as

Inhibition activity (%) = 100% × [(AS - ASB) /(AC -ACB)]

where AS, ASB, AC and ACB are the absorbance of sample, sample blank, control and control blank, respectively. The measurement was performed in triplicates. For kinetic analysis of AGH inhibition, a certain concentration of 0.6 U/mL AGH and different contents of sample compounds were incubated with a series of concentrations of substrate. The inhibitory kinetics of the investigated compounds was analyzed using the Lineweaver-Burk plot, double-reciprocal plot of the substrate concentration and velocity.

## Results and Discussion

*Inhibitory effects of 20 species of traditional Chinese herbal on α-glucosidase*

20 kinds of Chinese herbs were investigated for their α-glucosidase inhibitory. Among them, roots of *Cyperus rotundus *L. showed the best activity, just as shown in Table 1.

*Screening procedure*

The extract of *C*.* rotundus* was used as the real biological sample to carry out the affinity screening procedure, just as shown in Scheme 1. Firstly, α-glucosidase was immobilized on CNBr-activated sepharose beads, while blank supports were set as control. Secondly, immobilized α-glucosidase was incubated with 300 µL of *C*.

*rotundus *extracts to launch the affinity capture procedure. The highly-affinity components in *C*. *rotundus *could be specifically captured by the enzyme and fished out from the complex matrix. Thirdly, to characterize the captured compounds, the binders were released from immobilized α-glucosidase and subsequently separated and analyzed by UHPLC-QTOF MS. It was worth mentioning that not all the molecules retained on immobilized enzyme were of highly-affinity. Some of them might be non-specifically absorbed on the blank sepharose support. To eliminate this false positive, a parallel control was carried out throughout the whole screening procedure. By subtracting the signal of false positive, the highly-affinity ingredients could be recognized correctly, and these ingredients might be potent natural AGHI in *C*. *rotundus*.

*Screening results*

Screening results was shown in Figure 1. From the complex of *C*.* rotundus* extracts, peak 1, 2, 3, 4, 5 and 6 (Figure 1b, solid line) were captured by the immobilized enzyme. By subtracting the signal of “false positive” (Figure 1b, dotted line), the highly-affinity binders of α-glucosidase were finally recognized as peak 1-5 (Figure 1c). The structures represented by peak 1-5 were then characterized as cyperusphenol A (1), mesocyperusphenol A (2), cyperusphenol D (3), scirpusins B (5) and scirpusins A (5) by QTOF accurate MS spectrum (Figure 1d-1h) and comparing retention times with their standards. The standards preparation was described as *Isolated procedure of 5 natrual AGHIs from C. Rotundus* in *Experimental* section. (11). The above five molecules were believed to be possible tight-binding AGHIs in *C*.* rotundus*. It is worth noting that peak 6 (sugetriol (12), a typical sesquiterpene components in *C*.* rotundus*) would probably have been recognized as highly-affinity binder (Figure 1b) if there was no parallel control investigation. By comparison of the signal of blank support, sugetriol was finally recognized as “fault positive”, which absorbed only on the support matrix.

*Validation of screening results*

Although compound 1-5 were recognized as highly-affinity binders of α-glucosidase, these molecules still needed to confirm their α-glucosidase inhibitory activity. That is because not all the binders bound to the enzyme are inhibitors, some of them are just “frequent hitters (13) and unselectively clogging the protein by hydrophobic interaction without any inhibitory. To verify their inhibitory, the five compounds were isolated and purified from the *C*.* rotundus* extracts by open column spectrometry and Prep-HPLC, and subsequently determined their anti-α-glucosidase activity by traditional enzyme inhibition assay. As shown in Table 2, all these five tight-binding ligands were proved to be potent α-glucosidase inhibitors, with IC_50_ values of 1.18-21.4 m M, while IC_50_ value of reference drug acarbose was 1470 mM. The results suggested that these highly-affinity binders are really very strong natural AGHIs.

To validate the inhibitors affinity, the equilibrium dissociation constant (Ki) of these five compounds were investigated (Table 1). All these five ingredients showed strong affinity to the enzyme, with Ki values of 8.63-136 mM determined by Lineweaver-Burk plots (14) (Figure 2). Furthermore, the Lineweaver-Burk plots indicated that all of the five inhibitors were non-competitive inhibitors. The above results demonstrated that the screening procedure was performed by the affinity principle.

## Conclusion

The roots of *C. rotundus *have been proved to have antidiabetic activity in animal models. Aqueous ethanol extract of *C. rotundus *significantly lowered blood glucose level in alloxan-induced hyperglycemic rats (15). Stilbenoids dimers, such as scirpusin B (**4**) and scirpusin A (**5**), have been reported to be natural *α*-glucosidase inhibitors in *C. rotundus *(16). Our research successfully screened out the reported two stilbenoids dimmers. What’s more, by using this rapid affinity-based AGHI screening model, three stilbenoids trimers (i.e., cyperusphenol A, mesocyperusphenol A, cyperusphenol D) were fished out and identified as potent natural AGHIs. As far as we know, it was the first report about the α-glucosidase inhibitory of these three stilbenoids trimers. As shown in Table 1, inhibitory activity of the three stilbenoids trimers were about 10 fold higher than that of dimers. The activity difference between trimers and dimers might be derived from the different number of hydroxyl groups, which is in agreement with previous reports of structure-activity relationship of stilbenoids oligomers (17). Thus, the higher the number of free phenolic hydroxyl groups in a polystilbene core, the higher the α-glucosidase inhibitory.

In conclusion, an effective and rapid affinity fishing-based method was established for screening of alpha-glucosidase inhibitors from *C. rotundus *extract directly. Within a couple of hours, 5 natural active ingredients (i.e., (E)-cyperusphenol A, mesocyperusphenol A, cyperusphenol D, scirpusins B and scirpusins A) were rapidly fished out from 0.4 mL of *C. rotundus *extracts. The affinity captured compounds were then isolated by affinity elution and characterized by UHPLC-QTOF-MS as stilbenoid oligomers. These screened captured compounds were all proved to be potent alpha-glucosidase inhibitors by inhibitory assay *in *-*vitro*. Notably, the inhibitory activity of 3 stilbenoid timers was reported for the first time.This method could be modified and have the potential for rapidly screening of active compounds extracts against some new targets by immobilizing some other biomacromolecules.

## Conflict of interest

The authors have no conflict of interest to declare.
